# Manejo de Taquicardias de Complexo QRS Alargado na Sala de Emergência: O Que Realmente Importa

**DOI:** 10.36660/abc.20230829

**Published:** 2024-07-01

**Authors:** José Nunes de Alencar, Guilherme Dagostin de Carvalho, Renan Teixeira Campelo, Sandro Pinelli Felicioni, Matheus Kiszka Scheffer, Mariana Nogueira De Marchi

**Affiliations:** 1 Instituto Dante Pazzanese de Cardiologia São Paulo SP Brasil Instituto Dante Pazzanese de Cardiologia, São Paulo, SP – Brasil

**Keywords:** Eletrocardiografia, Taquicardia Ventricular, Arritmias Cardíacas, Serviço Hospitalar de Emergência

## Introdução

Estudos demonstraram que 80% das taquicardias de complexo QRS alargado são taquicardias ventriculares (TV). Esse número sobe para 90% quando há doença cardíaca estrutural subjacente.^1-4^ Existem critérios para diferenciar a TV da taquicardia supraventricular (TSV) que são importantes na prática clínica, mas não necessariamente com o propósito de fornecer um diagnóstico exato em cenários agudos.^5-9^

**Figura 1:**
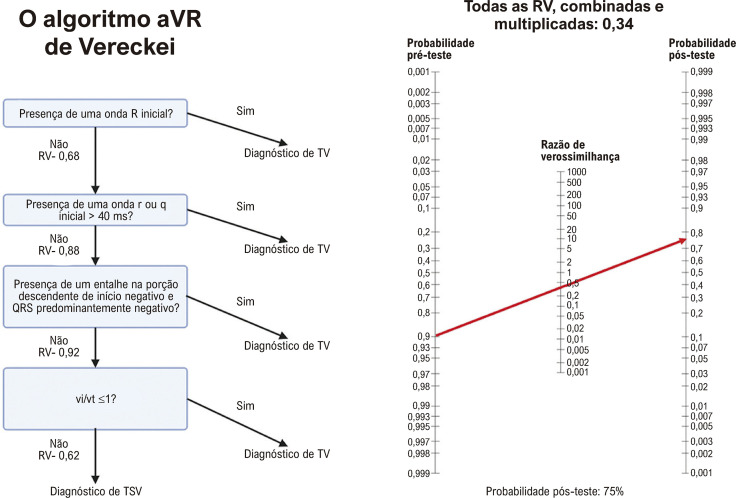
– Aplicação do raciocínio bayesiano para um paciente com taquicardia de complexo QRS alargado. Nomograma de Fagan ilustrando a probabilidade pós-teste de taquicardia ventricular. Mesmo com todos os critérios de Vereckei indicando “negativo”, permanece uma probabilidade de 75% de taquicardia ventricular. RV: razão de verossimilhança; TSV: taquicardia supraventricular; TV: taquicardia ventricular.

Embora os critérios tenham sido validados, eles não são tão eficazes na exclusão dessas condições como se poderia pensar. Um estudo de mundo real mostrou que os critérios de Vereckei tinham uma razão de verossimilhança negativa de 0,34.^10^ Isso significa que a aplicação desses critérios a um paciente com uma probabilidade pré-teste de 90% ainda resultaria em uma probabilidade pós-teste de 75% para TV (Figura 1).^11^ No mesmo estudo, os critérios de Brugada produziram uma razão de verossimilhança negativa semelhante a 0,24, resultando em uma probabilidade de 68% para TV.^10^ Outro motivo para não depender apenas desses critérios é a ausência de dados basais de eletrocardiograma (ECG). Por exemplo, um paciente com doença cardíaca congênita pode ter um eixo noroeste e outros achados únicos. Da mesma forma, um paciente com bloqueio das fibras de Purkinje e zonas eletricamente inativas pode apresentar características de ECG enganosas.^12,13^ Somando-se à complexidade, a TV idiopática, como a TV fascicular, muitas vezes apresenta padrões de ECG que imitam bloqueios de ramo ou fasciculares, levando a interpretações equivocadas.^14^ Outro caso emblemático é a TV por reentrada de ramo, onde o ECG durante a taquicardia apresentará critérios morfológicos de bloqueio de ramo esquerdo ou direito, confundindo ainda mais o diagnóstico.^15^

Ainda mais importante, a aplicação desses critérios não deve alterar o manejo clínico imediato. Tratar um paciente com TV como se tivesse TSV com uso de medicamentos antiarrítmicos com efeitos inotrópicos negativos, como bloqueadores dos canais de cálcio, pode levar à deterioração hemodinâmica. Em uma série, isso ocorreu em 100% dos pacientes tratados desta forma.^2^

Diante de um paciente com taquicardia de complexo QRS alargado, a prioridade é avaliar o estado clínico do paciente. Se o paciente apresentar sinais de deterioração clínica, como dor, dispneia ou sinais de choque, a cardioversão elétrica sincronizada imediata é a ação recomendada, de acordo com as diretrizes estadunidenses e europeias.^16,17^ A aplicação de algoritmos pode distrair os profissionais de saúde de focar no paciente, principalmente aqueles com menos experiência.^18^

**Figura 2:**
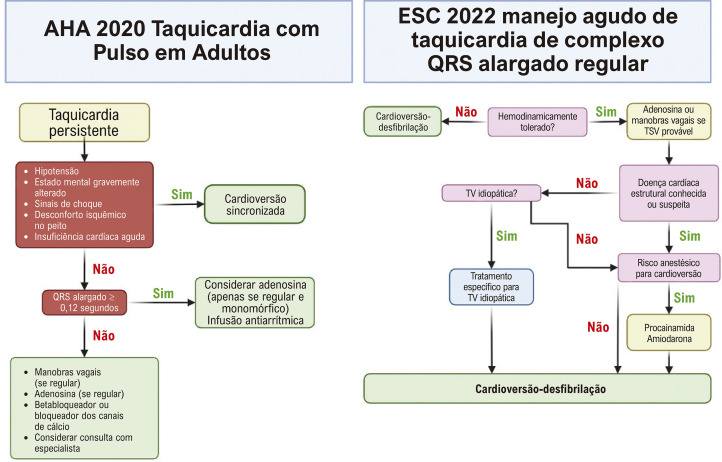
– Algoritmos para manejo de taquicardia. Algoritmos da American Heart Association (AHA) 2020 e da Sociedade Europeia de Cardiologia (ESC) 2022 para o manejo de taquicardias, incluindo as taquicardias de complexo QRS alargado, destacando a ausência de aplicação de critérios eletrocardiográficos no processo de tomada de decisão. TSV: taquicardia supraventricular; TV: taquicardia ventricular.

Em pacientes com taquicardia de complexo QRS alargado e estabilidade hemodinâmica, é crucial seguir as diretrizes nacionais relevantes sobre arritmia de emergência para manejo subsequente. Nos Estados Unidos, as diretrizes recomendam um teste de adenosina, se o tempo permitir. Esse teste tem dois propósitos: primeiro, a adenosina pode efetivamente terminar algumas TSV com aberrância; segundo, algumas formas de TV também podem responder à adenosina. Se o teste não for realizado ou se mostrar ineficaz, são recomendados antiarrítmicos intravenosos, como procainamida ou amiodarona. As diretrizes europeias, por outro lado, preconizam a cardioversão elétrica sincronizada para taquicardias de complexo QRS alargado, mesmo em pacientes hemodinamicamente estáveis, desde que o risco anestésico seja baixo.^17^ Notavelmente, nenhuma das diretrizes inclui uma etapa para a aplicação de critérios de diferenciação. A Figura 2 resume essas diretrizes.

Após a estabilização do paciente, uma discussão aprofundada com o cardiologista e o eletrofisiologista do hospital pode ser de valor inestimável.^19^ Nesse cenário, sua importância é mais sutil e reside no planejamento eletrofisiológico. Quando são utilizados em conjunto com os dados clínicos do paciente e resultados de outros exames diagnósticos, por exemplo, ecocardiograma, ressonância magnética cardíaca e estudos eletrofisiológicos, esses critérios podem servir como indicadores para o diagnóstico final e decisivo. Isso, por sua vez, pode orientar opções de tratamento que variam desde medicação ou ablação em casos mais simples até a implantação de cardioversor-desfibrilador. A Tabela 1 resume recomendações racionais baseadas em nosso ponto de vista.

**Tabela 1:** – Resumo das recomendações para taquicardias de complexo QRS alargado

Seguir as diretrizes nacionais relevantes para arritmia de emergência diante de um paciente com taquicardia de complexo QRS alargado.
Reservar o uso de critérios de diferenciação de TV e TSV para planejamento eletrofisiológico em vez de manejo clínico imediato.
Não confiar apenas nos critérios do ECG para diagnóstico; considerar os dados clínicos do paciente e outros exames diagnósticos, como ecocardiogramas e RMC.
Ter cuidado ao interpretar ECGs sem dados basais, especialmente em pacientes que têm uma doença congênita conhecida ou doença cardíaca estrutural.
Se o paciente apresentar sinais de deterioração clínica, proceder imediatamente à cardioversão elétrica sincronizada, conforme recomendado pelas diretrizes estadunidenses e europeias.
Consultar o eletrofisiologista do hospital para uma avaliação abrangente pós-estabilização, incorporando os critérios de ECG como uma evidência entre muitas.

*ECG: eletrocardiograma; RMC: ressonância magnética cardíaca; TSV: taquicardia supraventricular; TV: taquicardia ventricular.*

## Conclusão

A capacidade de diferenciar entre TV e TSV usando critérios de ECG, embora valorizada academicamente, pode não ser tão clinicamente impactante como tradicionalmente se acredita. A aplicação de apenas esses critérios pode levar a diagnósticos equivocados, sem alterar significativamente o manejo clínico imediato. É essencial seguir as diretrizes nacionais relevantes sobre arritmia de emergência e consultar um eletrofisiologista para uma avaliação abrangente pós-estabilização.
