# Supplementary material for: Manejo de Taquicardias de Complexo QRS Alargado na Sala de Emergência: O Que Realmente Importa
Source: Arq Bras Cardiol. 2024 Jul 1;121(6):e20230829. [Article in Portuguese] doi: 10.36660/abc.20230829 (PMC12080608; doi:10.36660/abc.20230829)
Supplement: Translation

# Translation: Wide QRS Tachycardias Management in Emergency Departments: What Really Matters

**DOI:** 10.36660/abc.20230829i

**Authors:** José Nunes de Alencar, Guilherme Dagostin de Carvalho, Renan Teixeira Campelo, Sandro Pinelli Felicioni, Matheus Kiszka Scheffer, Mariana Nogueira De Marchi

**Affiliations:** 1 Brazil Instituto Dante Pazzanese de Cardiologia, São Paulo, SP – Brazil

**Keywords:** Electrocardiography, Ventricular Tachycardia, Arrhythmias, Cardiac, Hospital Emergency Service

## Introduction

Studies have shown that 80% of wide QRS tachycardias are ventricular tachycardias (VT). This number rises to 90% if there is underlying structural heart disease.^1-4^ Criteria for differentiating between VT and supraventricular tachycardia (SVT) exist, and they are important in clinical practice, but not necessarily for the purpose of providing an exact diagnosis in acute settings.^5-9^

Although the criteria have been validated, they are not as effective at ruling out conditions as one might think. A real-world study showed that Vereckei’s criteria had a negative likelihood ratio of 0.34.^10^ This means that applying these criteria to a patient with a 90% pre-test probability would still result in a post-test probability of 75% for VT (Figure 1).^11^ In that study, the Brugada criteria yielded a similar negative likelihood ratio of 0.24, resulting in a 68% probability for VT.^10^ Another reason not to rely solely on these criteria is the absence of baseline electrocardiogram (ECG) data. For instance, a patient with congenital heart disease may have a northwest axis and other unique findings. Similarly, a patient with Purkinje fiber blockage and electrically inactive zones may present with misleading ECG features.^12,13^ Adding to the complexity, idiopathic VT, such as fascicular VT, often present ECG patterns that mimic bundle branch or fascicular blocks, leading to misinterpretation.^14^ Another emblematic case is bundle-branch reentry VT, where the ECG during tachycardia will display morphological criteria of either left or right bundle branch block, further confounding the diagnosis.^15^

**Figure 1:** – Applying Bayesian reasoning to a patient with wide QRS tachycardia. Fagan’s nomogram illustrating the post-test probability of ventricular tachycardia. Even with all Vereckei’s criteria indicating ‘negative’, there remains a 75% likelihood of ventricular tachycardia. LR: likelihood ratio; SVT: supraventricular tachycardia; VT: ventricular tachycardia.

Most importantly, the application of these criteria should not alter immediate clinical management. Treating a patient with VT as if they had SVT using antiarrhythmic drugs with negative inotropic effects, such as calcium channel blockers, can lead to hemodynamic deterioration. In one series, this occurred in 100% of patients treated in this manner.^2^

When faced with a patient exhibiting wide QRS tachycardia, the priority is to assess the patient’s clinical status. If the patient shows signs of clinical deterioration, such as pain, dyspnea, or signs of shock, immediate synchronized electrical cardioversion is the recommended course of action, according to both American and European guidelines.^16,17^ The application of algorithms can distract healthcare providers, particularly those with less experience, from focusing on the patient.^18^

In patients with wide QRS tachycardia and hemodynamic stability, it is crucial to follow the relevant national guidelines on emergency arrhythmia for further management. In the United States, the guidelines recommend an adenosine test, if time permits. This test serves two purposes: first, adenosine can effectively terminate some SVT with aberrancy; second, some VT may also respond to adenosine. If the test is not performed or proves ineffective, intravenous antiarrhythmics, such as procainamide or amiodarone, are recommended. European guidelines, on the other hand, advocate for synchronized electrical cardioversion for wide QRS tachycardias even in hemodynamically stable patients, provided that the anesthetic risk is low.^17^ Noticeably, neither guideline includes a step for applying differentiation criteria. Figure 2 summarizes these guidelines.

**Figure 2:** – Tachycardia Management Algorithms. American Heart Association (AHA) 2020 and European Society of Cardiology (ESC) 2022 algorithms for the management of tachycardia and wide QRS complex tachycardias, highlighting the absence of electrocardiographic criteria application in the decision-making process. SVT: supraventricular tachycardia; VT: ventricular tachycardia.

After the patient has been stabilized, a thorough discussion with the hospital’s cardiologist and electrophysiologist can be invaluable.^19^ In this scenario, their importance is more nuanced and lies in electrophysiological planning. When used in conjunction with the patient’s clinical data and results from other diagnostic tests, for example, echocardiogram, cardiac magnetic resonance, and electrophysiological studies, these criteria can serve as indicators for the final and decisive diagnosis. This, in turn, can guide treatment options ranging from medication or ablation in simpler cases to the implantation of a cardioverter-defibrillator. Table 1 summarizes rational recommendations based on our point of view.

**Table 1:** – Summary of recommendations for wide QRS tachycardias

Follow relevant national guidelines on emergency arrhythmia when encountering a patient with wide QRS tachycardia.
Reserve the use of VT and SVT differentiation criteria for electrophysiological planning rather than immediate clinical management.
Do not rely solely on ECG criteria for diagnosis; consider the patient's clinical data and other diagnostic tests, such as echocardiograms and cardiac MRI.
Be cautious when interpreting ECGs without baseline data, especially in cases where the patient has a known congenital condition or structural heart disease.
If the patient shows signs of clinical deterioration, proceed immediately to synchronized electrical cardioversion, as recommended by both American and European guidelines.
Consult with the hospital's electrophysiologist for a comprehensive evaluation post-stabilization, incorporating ECG criteria as one piece of evidence among many.

*ECG: electrocardiogram; MRI: magnetic resonance imaging; SVT: supraventricular tachycardia; VT: ventricular tachycardia.*

## Conclusion

The ability to differentiate between VT and SVT using ECG criteria, while academically valued, may not be as clinically impactful as traditionally believed. Relying solely on these criteria can lead to misdiagnoses and may not significantly alter immediate clinical management. It is essential to follow the relevant national guidelines on emergency arrhythmia and to consult with an electrophysiologist for a comprehensive evaluation post-stabilization.
